# Predicting recurrence of prostate cancer after radical treatment using AI models based on PET/CT radiomics: a dual-center study

**DOI:** 10.3389/fonc.2026.1733046

**Published:** 2026-04-06

**Authors:** Zhanxiong Yi, Weichun Li, Yueyang Lou, Qinbing Zhou

**Affiliations:** 1 Department of Nuclear Medicine, Dongyang People’s Hospital, Dongyang, Zhejiang, China; 2 Department of Nuclear Medicine, Quzhou People’s Hospital, Quzhou, Zhejiang, China

**Keywords:** deep learning, positron emission tomography/computed tomography, prostate cancer, radiomics, recurrence prediction

## Abstract

Predicting prostate cancer (PCa) recurrence after radical treatment is crucial for personalised adjuvant therapy. This study aimed to compare different algorithms in order to select the best model for predicting recurrence. Therefore, a retrospective cohort analysis was conducted on 72 patients with radical prostate cancer, including 39 patients with biochemical recurrence and 33 patients without recurrence. We extracted features from imaging data, construct and evaluate 10 machine learning models and 8 deep learning models. Model performance was assessed using the area under the curve (AUC), accuracy, sensitivity, specificity, precision, 10-fold cross-validation AUC, and F1-score. In addition, the feature importance was analysed. Among all models, the MLP-Mixed-Act model exhibited superior performance in all evaluation indicators (AUC = 0.910, accuracy =0.819, sensitivity =0.744, specificity =0.909, precision =0.912, F1 = 0.817), thereby indicating its strong predictive ability and clinical application potential. This study provides a theoretical basis for the development of preventive and non-invasive recurrence prediction tools. Especially in the context of valuing the tumor microenvironment, accurate recurrence prediction can effectively help select immunotherapy strategies, improve treatment efficacy and prognosis, and support for personalized treatment of PCa.

## 1 Introduction

Prostate cancer (PCa) represents the most prevalent malignant neoplasm within the male genitourinary system, with its incidence demonstrating a consistent upward trend annually (1). Standard therapeutic approaches for localized PCa include radical prostatectomy and radical radiotherapy, however, these interventions are associated with a persistent risk of post-treatment recurrence. Empirical evidence indicates that biochemical recurrence occurs in approximately 20% to 40% of patients following radical prostatectomy and in 30% to 50% of patients after radical radiotherapy, serving as a precursor to tumor progression characterized by clinical recurrence or metastasis (2). Consequently, a substantial proportion of patients ultimately experience clinical relapse or progress to castration-resistant prostate cancer, which is associated with a poor prognosis, reflected by a 5-year survival rate of merely 10% to 15%, and presents significant therapeutic challenges (3). Accurate recurrence prediction remains a major obstacle, limiting the ability to effectively implement personalized treatment strategies. Despite significant advances in treatment methods, the lack of precise non-invasive early recurrence detection methods has resulted in a significant gap in clinical practice.

Prostate-specific membrane antigen (PSMA), a transmembrane protein that is highly expressed in PCa cells, is closely linked to PCa recurrence and plays a critical role in the malignant progression of the disease (4). Imaging modalities targeting PSMA, particularly radionuclide-labeled PSMA PET/CT, have undergone rapid development and have fundamentally transformed the diagnostic and therapeutic landscape of PCa (5). Currently, PSMA-based positron emission tomography/computed tomography (PET/CT) imaging demonstrates significant potential in the diagnosis, staging, grading, prognosis, and monitoring of recurrence in PCa. It has been shown to enhance the localization of primary tumors (6) and improve the detection of recurrent PCa (7) in patients following radical prostatectomy, even at low prostate-specific antigen (PSA) levels (8). This imaging approach has gained clinical acceptance and has been incorporated into clinical guidelines, underscoring its importance as a noninvasive and precise tool for the diagnosis and prognostic evaluation of PCa. Despite these advances, the clinical utility of PSMA PET/CT for recurrence detection is still limited by its dependence on PSA levels, which may not be sensitive enough to detect early recurrence in certain patients. This limitation emphasizes the need for innovative imaging or image analysis methods that may provide more accurate recurrence detection.

Recently, the collaborative integration of big data technology and medical imaging diagnostic methods has led to a new imaging approach called radiomics. This method has significant clinical value as it can extract various features from medical images for the evaluation and prediction of important diseases such as tumors (9). Radiomics combined with artificial intelligence technologies (10), such as deep learning (DL) and machine learning (ML), extracts relevant information from a large amount of clinical data for analysis and prediction, making accurate clinical decisions for different patients, and has become a non-invasive and cost-effective strategy (11). In PCa research, Nanakaran et al. used T2 weighted MRT images to integrate ML technology to develop a model for predicting prostate cancer recurrence in patients after radiotherapy, with an area under the curve (AUC) value of 0.84 (12). In addition, Wang et al. developed a deep learning based radioprediction model for predicting biochemical recurrence in advanced prostate cancer patients using a pre-treatment epidemiological spread coefficient map (13). In addition, a recent review on the application of artificial intelligence (AI) in PCa comprehensively elucidates the key findings and trends in this field, further emphasizing the important role of AI, especially ML and DL algorithms, in promoting radiological assisted diagnosis, treatment, prognosis, and recurrence prediction of PCa (14–16).

In summary, the combination of AI and radiomics provides a potential non-invasive assessment method, laying the foundation for the advancement of innovative tools for disease risk prediction modeling. However, existing models still have certain limitations in accurately predicting recurrence after curative treatment. The goal of this study is to use radiomics data to find better AI models and improve the accuracy of predicting prostate cancer recurrence after radical treatment to compensate for this deficiency.

**Figure 1:**
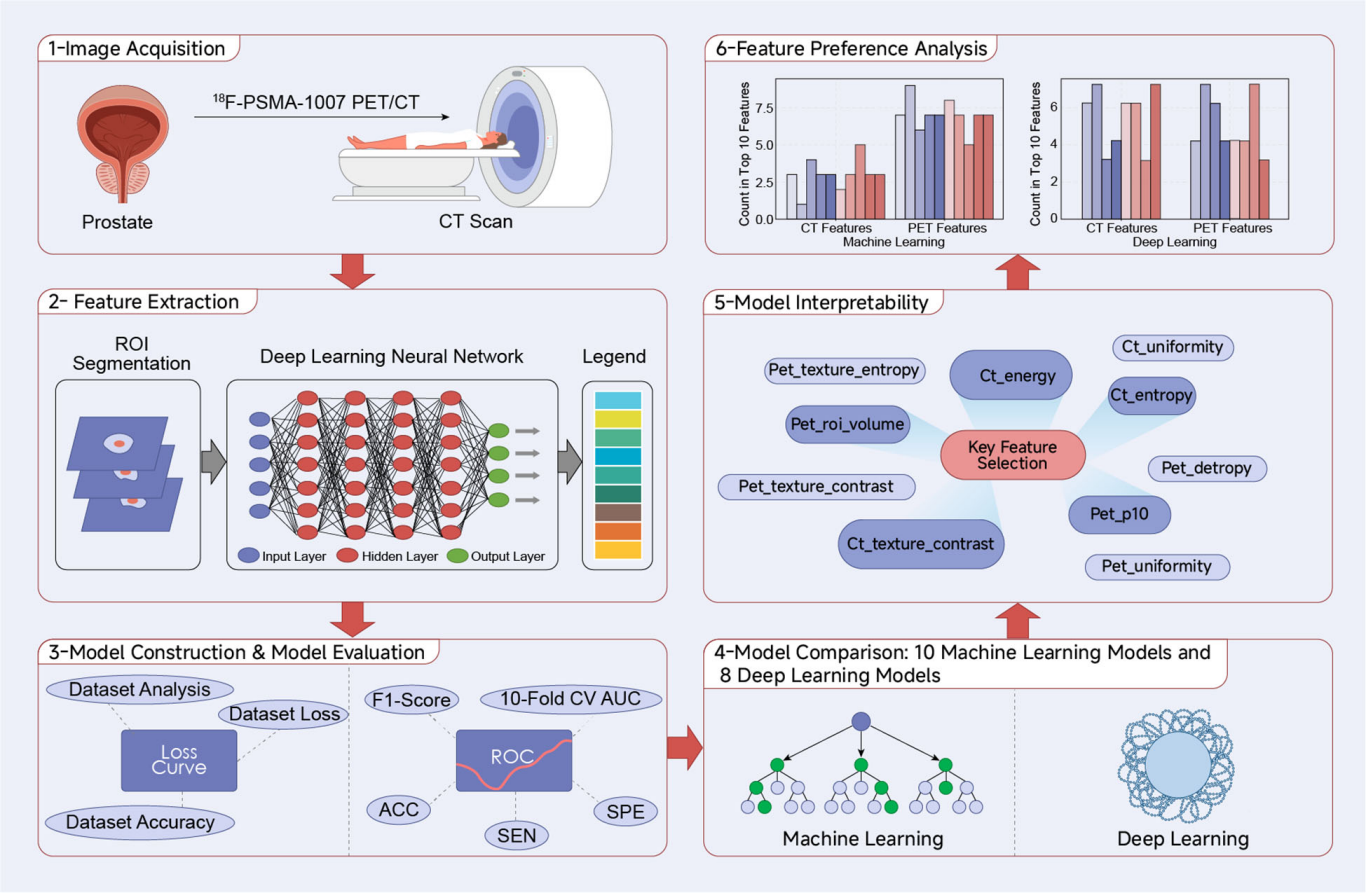
Trial chart.

In this study, we evaluated the performance of multiple ML and DL models in predicting the recurrence risk of patients after radical PCa treatment using indicators such as AUC, accuracy, sensitivity and characteristics, which provides potential and effective predictive tools for predicting the recurrence of PCa and offers more tools for formulating precise treatment strategies for PCa (**Figure 1**).

## 2 Methods

### 2.1 Patient population and study design

This retrospective dual-center study was conducted in accordance with the principles of the Declaration of Helsinki and was approved by the Institutional Review Board (IRB) of Dongyang People’s Hospital (IRB Approval No: SC-2025-115). Written informed consent was obtained from all participants.

**Figure 2:**
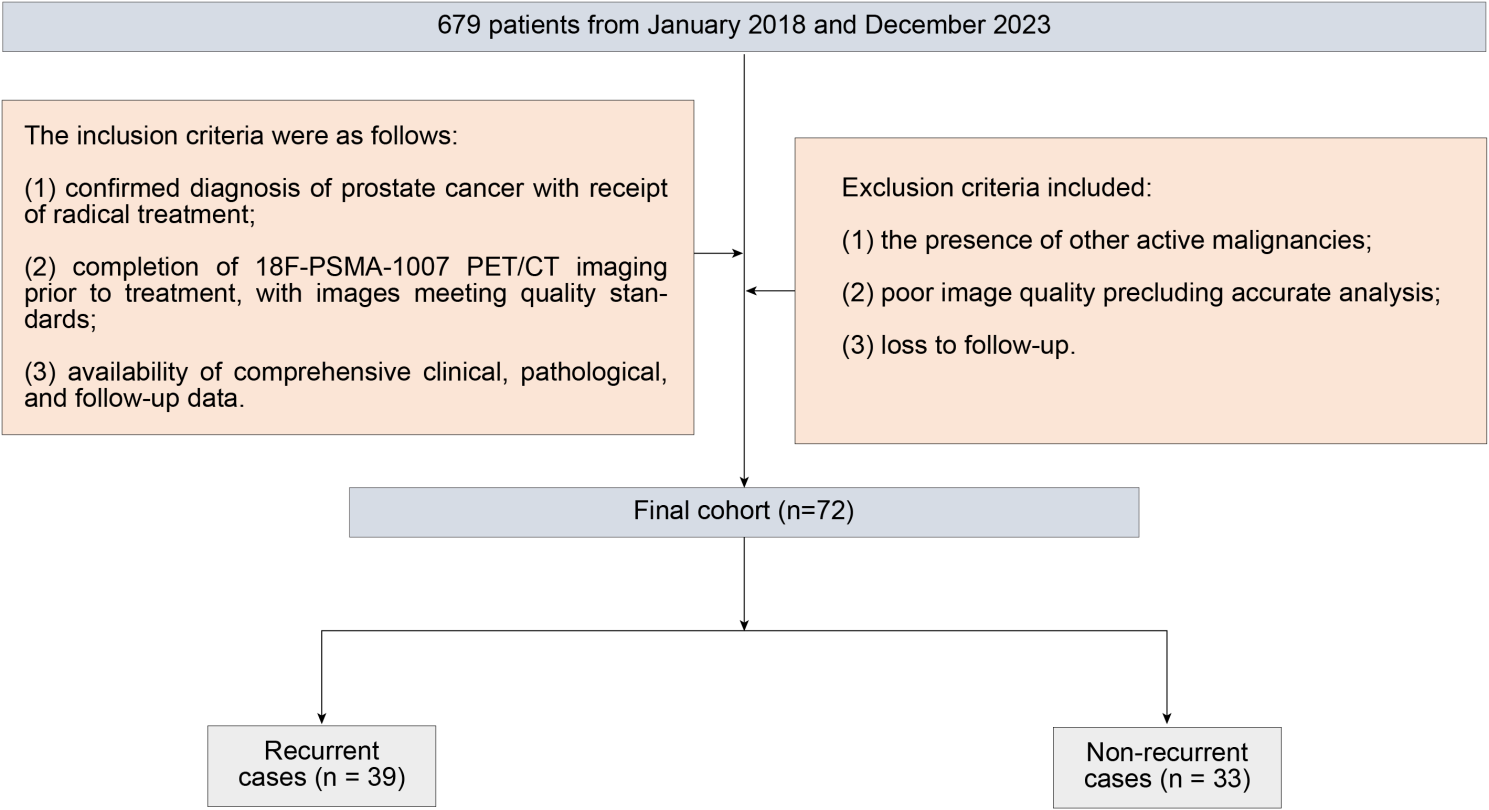
Patient screening.

We initially screened 679 patients with histologically proven prostate adenocarcinoma who underwent ¹^7^F-PSMA-1007 PET/CT between January 2018 and December 2023. The inclusion criteria were as follows: 1) confirmed diagnosis of PCa with receipt of radical treatment; 2) completion of ^18^F-PSMA-1007 PET/CT imaging prior to treatment, with images meeting quality standards; and 3) availability of comprehensive clinical, pathological, and follow-up data. Exclusion criteria included: 1) the presence of other active malignancies; 2) poor image quality precluding accurate analysis; and 3) loss to follow-up (**Figure 2**).

The final study population included 72 eligible patients, with 39 patients experiencing relapse and 33 patients not experiencing relapse. The primary endpoint was biochemical recurrence, defined as a confirmed PSA level ≥0.2 ng/mL followed by a subsequent rise.

### 2.2 Image acquisition and reconstruction

All ¹^7^F-PSMA-1007 PET/CT scans were performed on Siemens Biograph Vision PET/CT and GE Discovery max+ PET-CT. Patients received an intravenous injection of ¹^7^F-PSMA-1007 (radiochemical purity >95%) at a dose of 3.7 MBq/kg. After an uptake period of 60 ± 5 minutes, PET images were acquired from the mid-thigh to the skull base using a 3D acquisition mode. Images were reconstructed using an ordered-subset expectation maximization (OSEM) algorithm (4 iterations, 16 subsets) with time-of-flight (TOF) and point-spread-function (PSF) corrections.

A low-dose CT scan was performed for attenuation correction and anatomical localization with the following parameters: tube voltage 120 kV, tube current 40–100 mAs (automated modulation), slice thickness 1.25 mm, and a reconstruction matrix of 512×512. Adaptive statistical iterative reconstruction (ASIR-V) was applied at a 40% weight to minimize artifacts. All raw DICOM images were converted to NIfTI-1 format (.nii.gz) for subsequent analysis.

### 2.3 Image preprocessing and tumor segmentation

Image Registration and Standardization: Rigid registration of PET and CT images was performed using the Elastix module in 3D Slicer (v5.6.0), with mutual information as the similarity metric, achieving a spatial alignment error of <1 mm. Standardized uptake values were calculated based on body weight using MIM Software (v7.8). Physiological uptake in organs such as the bladder and salivary glands, standard uptake value (SUV) <2.5 without anatomical correlation, was manually masked.

Manual Segmentation (Ground Truth): Volumes of interest (VOIs) encompassing the primary prostate lesions and metastatic foci were manually delineated on the fused PET/CT images by two nuclear medicine physicians with 8 years of experience using ITK-SNAP (v3.8.1), and intraclass correlation coefficient (ICC) was calculated to evaluate the segmentation consistency. All radiomic features were extracted with fixed parameter settings (fixed bin width, resampling voxel size, and image normalization method) to avoid variability from parameter tuning. Features with poor stability (ICC < 0.75) were excluded in the preprocessing step. The feature selection procedure (including univariate analysis and LASSO regression) was performed under a fixed random seed to guarantee repeatability of the modeling process. The resulting 3D VOIs were saved as binary label maps (.nii.gz).

Semi-Automated Segmentation: To improve efficiency for the large dataset, a pre-trained 3D nnU-Net model (v2.2.1) was integrated into ITK-SNAP via a Python plugin. The model, trained on manually annotated cases, generated initial VOIs automatically. These were reviewed and manually corrected by experts to ensure a minimum dice similarity coefficient (DSC) of 0.85 compared to the ground truth.

### 2.4 Radiomics feature extraction and selection

All patients’ PET and CT images underwent standardized preprocessing, with features standardized using Z-score normalization. High-dimensional features, including shape, intensity, texture, and other characteristics were extracted using an imaging omics platform. A total of 1734 radiomic features were initially extracted using Pyradiomics based on the ROIs delineated with ITK-SNAP. To avoid overfitting and reduce dimensionality, a three-step feature selection was performed (1): Reproducibility filtering: Features with intraclass correlation coefficient (ICC) < 0.75 were excluded to ensure the reliability of segmentation and feature extraction (2); Univariate statistical test: Features with P > 0.05 (Mann-Whitney U test) were removed (3); LASSO regression with 10-fold cross-validation: The least absolute shrinkage and selection operator (LASSO) algorithm was applied to select the optimal feature subset, where the tuning parameter λ was determined by the minimum cross-validation error. Finally, 146 radiomic features with non-zero coefficients were retained for subsequent model construction, which effectively balanced the model performance and computational efficiency. A total of 146 radiomics features were extracted from each VOI using PyRadiomics (v3.0.1) (17) following the Image Biomarker Standardization Initiative (IBSI v2) (18). Features included first-order statistics (n=19), texture features (n=113) from gray-level co-occurrence matrices (GLCM) and gray-level run-length matrices (GLRLM), and morphological features (n=14). Prior to extraction, images were resampled to an isotropic voxel size of 2×2×2 mm³ and discretized with 32 gray-level bins. Regarding the assessment of feature stability, the three segmentations were generated as follows: three repeated manual segmentations were performed by the same expert nuclear medicine physician, with each segmentation session separated by a minimum two-week interval to minimize recall bias. This intra-observer test-retest approach was designed to isolate and quantify the variability inherent to the manual delineation process itself. The intraclass correlation coefficient (ICC) was then calculated across these three repeated segmentations for each of the 72 randomly selected cases. Features demonstrating high consistency (ICC > 0.85) across this manual delineation variability were considered stable and retained for subsequent analysis. Feature selection was performed during the cross validation cycle, removing redundant features (Spearman’s |ρ| >0.7) and retaining features with high variance.

### 2.5 Machine learning framework and model configurations

We implemented a comprehensive ML framework using 10 distinct algorithms through the caret package in R (version 4.1). The models included: Logistic Regression (GLM with binomial family), Random Forest (RF with tuneLength=5), Support Vector Machine (SVM Radial with probability=TRUE), Decision Tree (DT), K-Nearest Neighbors (KNN), Neural Network (NN), NaiveBayes, Linear Discriminant Analysis (LDA), Elastic Net (GLMNet), and Conditional Inference Tree (CTree). All models were optimized for AUC (Area Under the Curve) as the primary performance metric. Class imbalance was mitigated using inverse-frequency class weighting. Specifically, the class weight for each category was calculated as the inverse of its sample frequency in the training set.This method automatically assigns higher weights to minority classes and lower weights to majority classes, preventing the model from being biased toward the more frequent class. By introducing inverse-frequency class weighting into the loss function, we effectively alleviated the adverse effects of class imbalance, improved the recognition ability of the model for underrepresented samples, and enhanced the generalization and robustness of the final model.

### 2.6 Deep learning architecture and training

For DL analysis, we developed 8 distinct Multi-Layer Perceptron (MLP) architectures using the keras framework with TensorFlow backend. The configurations included: Basic MLP (MLP-Basic, 64–32 ReLU layers), Deep MLP (MLP-Deep, 128-64-32–16 ReLU layers), Dropout-regularized MLP (MLP-Dropout, with 30% dropout), Mixed-activation MLP (MLP-Mixed-Act, tanh-ReLU-SeLU-ReLU), RMSprop-optimized MLP (MLP-RMSprop), Small-batch MLP (MLP-Small-Batch, batch_size=8), and Wide architecture MLP (MLP-Wide, 256-128–64 units). All models used Adam optimizer, binary cross-entropy loss, sigmoid output activation, and were trained for 50 epochs with early stopping (patience=10) and 30% validation split. To address the class imbalance, all DL models were trained using a weighted binary cross-entropy loss.

### 2.7 Model training and validation strategy

All models employed a consistent 10-fold cross-validation with 3 repeats for hyperparameter tuning and performance estimation. For DL models, we implemented checkpoint saving to enable resumable training and prevent data loss.

### 2.8 Feature importance analysis

Feature importance was quantified using permutation importance analysis, where each feature was randomly shuffled multiple times (n= 5 repeats) and the corresponding decrease in model performance (AUC reduction) was measured. Top-10 important features were identified for each model, and comprehensive frequency analysis across all models was performed to determine consistently important radiomics features.

### 2.9 Performance evaluation metrics

Model performance was comprehensively evaluated using multiple metrics: AUC, accuracy, sensitivity, specificity, precision and F1-score. Statistical significance was assessed through repeated cross-validation. Receiver Operating Characteristic (ROC) curves were generated with smoothing enabled, and all metrics were calculated on the full dataset to provide comprehensive model comparisons.

### 2.10 Statistical software

All statistical analyses were performed using R software (v4.4.0). Key packages included irr (v0.84.1) for ICC, glmnet (v4.1-8) for LASSO, pROC (v1.18.5) for ROC analysis, and survival (v3.5-7) and survminer (v0.4.9) for survival analysis. Python (v3.10) with PyTorch (v2.1.0) and PyRadiomics (v3.0.1) was used for DL and feature extraction. Figures were generated using ggplot2 in R and matplotlib in Python.

## 3 Results

### 3.1 Patient characteristics

The final study population included 72 eligible patients. Among them, 39 patients experienced biochemical recurrence, while 33 patients had no evidence of disease recurrence (**Figure 2**). **Table 1** provides a detailed list of patient comprehensive characteristics

**Table 1:** Patients.

Project	Total	Non-Recurrent	Recurrent	t/(^2^	*P*
	72	33	39		
Age	73.1 ± 8.0	71.8 ± 6.7	74.2 ± 8.9	1.59	0.211
Weight	67.2 ± 10.0	69.1 ± 11.3	65.6 ± 8.6	2.12	0.15
^18^F	273.4 ± 69.8	289.6 ± 53.5	259.3 ± 79.3	3.44	0.068
PSA value	0.7 (0.0, 9.9)	0.0 (0.0, 0.2)	4.7 (0.8, 24.5)	26.12	<0.001
FPSA	0.2 (0.0, 1.4)	0.0 (0.0, 0.0)	0.6 (0.2, 8.7)	24.87	<0.001
Pathological type				2.22	0.329
Prostatic ductal adenocarcinoma	1 (1.4)	1 (3.1)	0 (0.0)		
Prostatic adenocarcinoma	22 (31.9)	12 (37.5)	10 (27.0)		
Prostatic acinar adenocarcinoma	46 (66.7)	19 (59.4)	27 (73.0)		
Gleason score				16.26	0.039
2 + 2	1 (1.8)	0 (0.0)	1 (3.7)		
3 + 3	5 (9.1)	5 (17.9)	0 (0.0)		
3 + 4	13 (23.6)	8 (28.6)	5 (18.5)		
4 + 3	11 (20.0)	7 (25.0)	4 (14.8)		
4 + 4	11 (20.0)	6 (21.4)	5 (18.5)		
4 + 5	4 (7.3)	0 (0.0)	4 (14.8)		
5 + 3	1 (1.8)	0 (0.0)	1 (3.7)		
5 + 4	6 (10.9)	2 (7.1)	4 (14.8)		
5 + 5	3 (5.5)	0 (0.0)	3 (11.1)		
WHOISUP grade	3 (5.4)	0 (0.0)	3 (11.1)		
1				11.14	0.025
2	5 (9.6)	5 (18.5)	0 (0.0)		
3	15 (28.8)	10 (37.0)	5 (20.0)		
4	8 (15.4)	4 (14.8)	4 (16.0)		
5	13 (25.0)	6 (22.2)	7 (28.0)		

### 3.2 Machine learning-based prediction of prostate cancer recurrence

**Figure 3:**
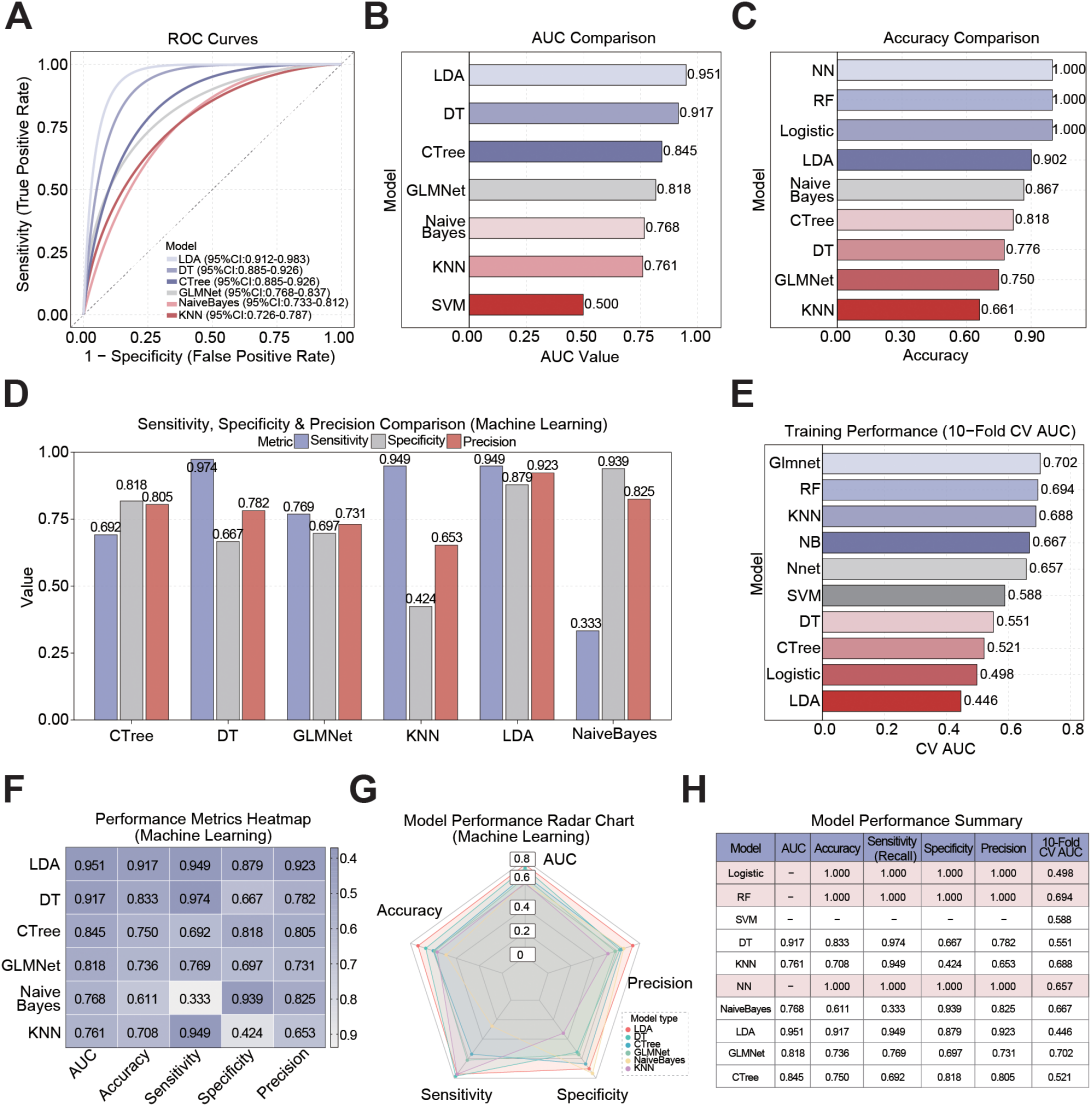
Performance evaluation of 10 ML models in PCa recurrence prediction. **(A)** ROC curves of 10 binary classification models based on a complete standardized dataset. **(B)** Comparison of the AUC values for ML models. **(C)** Comparison of the accuracy of the 10 ML models. **(D)** Comparison of the sensitivity, specificity and precision of ML models. **(E)** Comparison of the 10-fold cross-validation AUC value of ML models. **(F)** Heat map of four main performance indicators: AUC, accuracy, sensitivity, precisaion, and specificity. The color gradient ranges from light (low) to dark (high) to reflect numerical changes in these indicators. **(G)** Radar chart for comprehensive evaluation of four standardized performance indicators (AUC, accuracy, sensitivity, specificity and precision) of multiple models. **(H)** Summary of the core performance indicators of the 10 models based on prediction results.

The goal of this study was to evaluate the performance of ten machine learning approaches when predicting return of PCa: GLM, RF, LDA, DT, SVM, CTree, GLMNet, NaiveBayes, NN, and KNN. **Figure 3A** shows when looking at ROC curves, LDA, DT, and CTree models all performed well and AUC values of 0.951, 0.845, and 0.818, respectively indicated GLMNet, DT, and LDA had good discriminative ability in distinguishing recurrent from non-recurrent samples. **Figure 3B** indicates the NaiveBayes, KNN, and SVM models did poorly; their AUC values were 0.768, 0.761, and 0.500, respectively. In general, GLM, RF, and NN models did not yield AUC values indicating these models could not differentiate recurrent and non-recurrent samples.

In the accuracy evaluation, CTree, LDA, and NaiveBayes scored well, with ACC scores of 0.818, 0.902, and 0.867, respectively. While DT, GLMNet, and KNN were less robust with slightly lower ACC scores (0.776, 0.750, 0.661), respectively. It is clear when we look at **Figures 3C**, the NN, RF, and GLM models do not allow for meaningful accuracy numbers due to the lack of relevant ROCs. Model performance was further evaluated regarding detection of sample recurrence (i.e., sensitivity, specificity and precision). The LDA model again performed better among all the models, with a sensitivity of 0.949, specificity of 0.879 and precision of 0.923. Both DT and KNN models performed reasonably well with a sensitivity over 0.9 despite low specificity and precision (0.667 and 0.42 for DT, and 0.782 and 0.653 for KNN, respectively). Conversely, the NaiveBayes model failed to detect repeated cases and exhibit a substantial bias towards one category confirming low sensitivity (0.333) and high specificity and precision (0.939 and 0.825; **Figure 3D**). Then, we conducted three separate 10-fold cross validation tests to evaluate generalizability of the model. In **Figure 3E**, we observe the best performing model tested from all models was GLMNet, which had a 10-fold CV AUC of 0.702, followed by RF (0.694), KNN (0.684), and NaiveBayes (0.667). The 10-fold CV-AUC of LDA was merely 0.446, indicating potential stability issues and limited applicability to new datasets, even though it performed quite well on our dataset (AUC = 0.951).

The study results were displayed in an extensive manner by using performance heatmaps and radar charts. Each model’s performance was assessed according to five indicators: AUC, accuracy, sensitivity, specification and precision. Overall, the LDA model had the best performance overall across all metrics, where the DT came a close second, retaining largely equivalent performance across performance metrics. In **Figures 3F**, notice that the metrics indicate the NaiveBayes and KNN models performed the worst, with the CTree and GLMNet models following closely behind with pretty low accuracy. The radar chart provides additional evidence of this result, demonstrating that the LDA model is the most balanced and has the widest coverage in four-dimensional performance. The DT model ranks second, while models from GLMNet and CTree demonstrate a notable decrease in accuracy, and models from NaiveBayes and KNN have notable shortcomings (**Figure 3G**). You can see a summary of how well each machine learning model predicted PCa recurrence in **Figure 3H**. Each model’s performance differed significantly across several metrics, according to the results. With an AUC of 0.951, accuracy of 0.917, sensitivity of 0.949, specificity of 0.879 and precision of 0.923, the LDA model accomplishes the best results on the dataset. But LDA’s cross-validation AUC of just 0.446 suggests it has little generalizability and could be dangerous. With an AUC of 0.818, accuracy of 0.736, sensitivity of 0.769, specificity of 0.697 and precision of 0.731, GLMNet, on the other hand, shows a better balanced performance across measures. Notably, out of all the models, GLMNet has the highest 10-fold CV AUC at 0.702, which suggests that it is quite good at generalizing.

### 3.3 Feature importance in ML models

**Figure 4:**
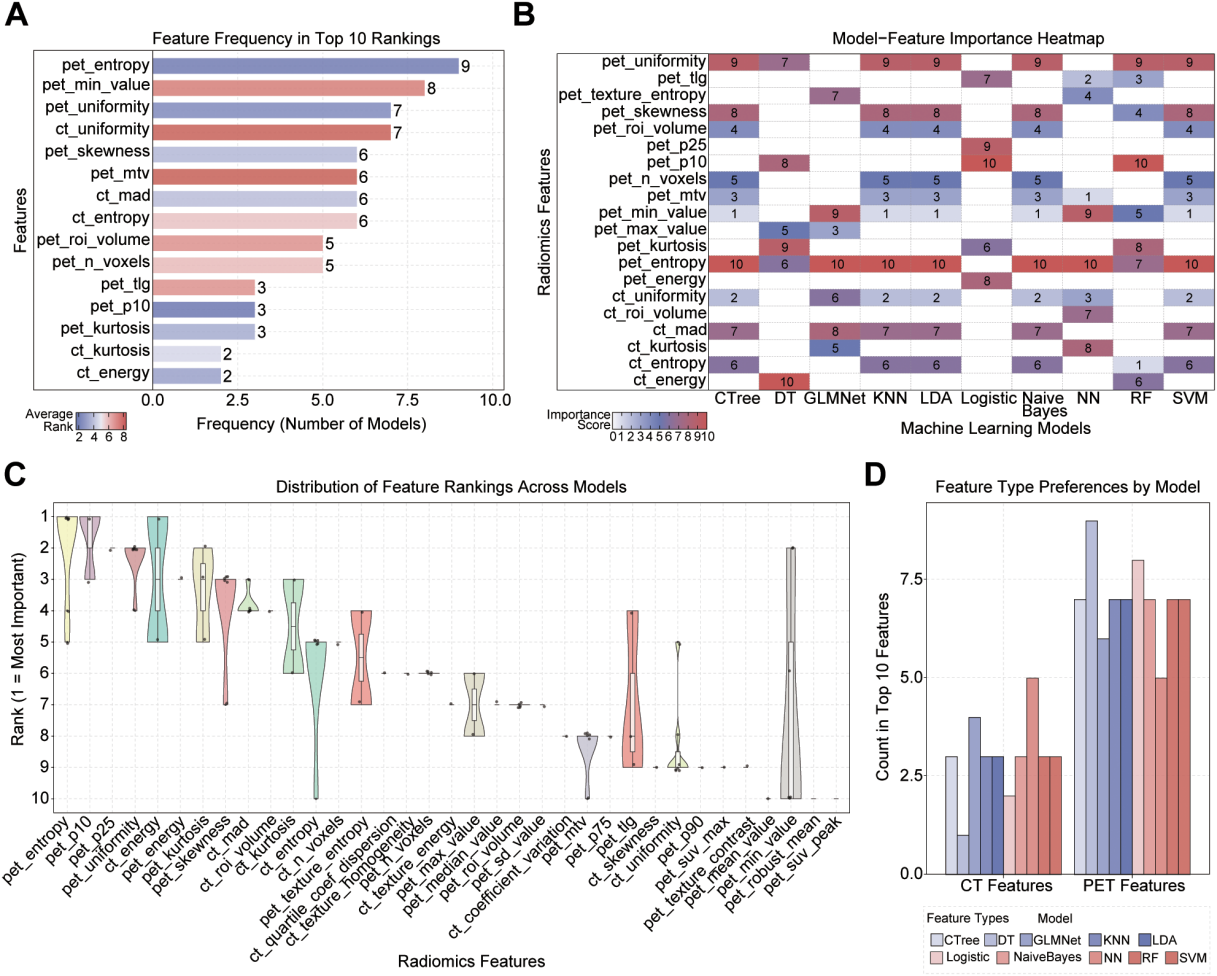
Importance analysis and preference comparison of features in ML models. **(A)** Feature frequency statistics. The color of each bar represents the average ranking of the feature in the selected model, with darker colors (red) indicating higher frequency. **(B)** Feature importance heatmap, where darker colors (red) correspond to greater importance. **(C)** Distribution of feature rankings across different models. Features are ranked by median, with the most stable and important features positioned at the top. **(D)** Statistics on model preferences for feature types.

We systematically analyzed the feature selection preferences of ML models to explain their feature selection preferences in predicting recurrence. Frequency analysis shows that pet_ entropy is the highest frequency selected feature (9 times), highlighting its significant contribution to prediction. Then there are pet_invalue (8 times), pet_uniformy, and ct_uniformy (7 times each). Frequency statistics indicate that although some CT features are representative, overall, PET features dominate in ML model selection (**Figure 4A**). Next, we continued to explore the importance scores (**Figure 4B**) and stability distributions (**Figure 4C**) of these features in different ML models. The results showed that both pet_detropy and pet_uniformity have high average importance and stability distribution rankings, which means they are crucial for predicting recurrence in most ML models. On the contrary, the average importance and ranking of ct_deergy, ct_kurtosis, and pet_cin-value are relatively low and scattered, which means their contribution to predicting recurrence is not significant. In addition, although the overall performance of ct_energy and ct_mad is poor, they are outstanding in a single model and can be used as important features of specific models. Finally, we categorized and summarized the preferences of each model for PET and CT features, and found that almost all ML models preferred PET features (**Figure 4D**). This indicates that although different models have different ways of extracting information, overall, PET features have stronger universal predictive ability than CT features in ML models.

### 3.4 Performance comparison of deep learning models for predicting prostate cancer recurrence

**Figure 5:**
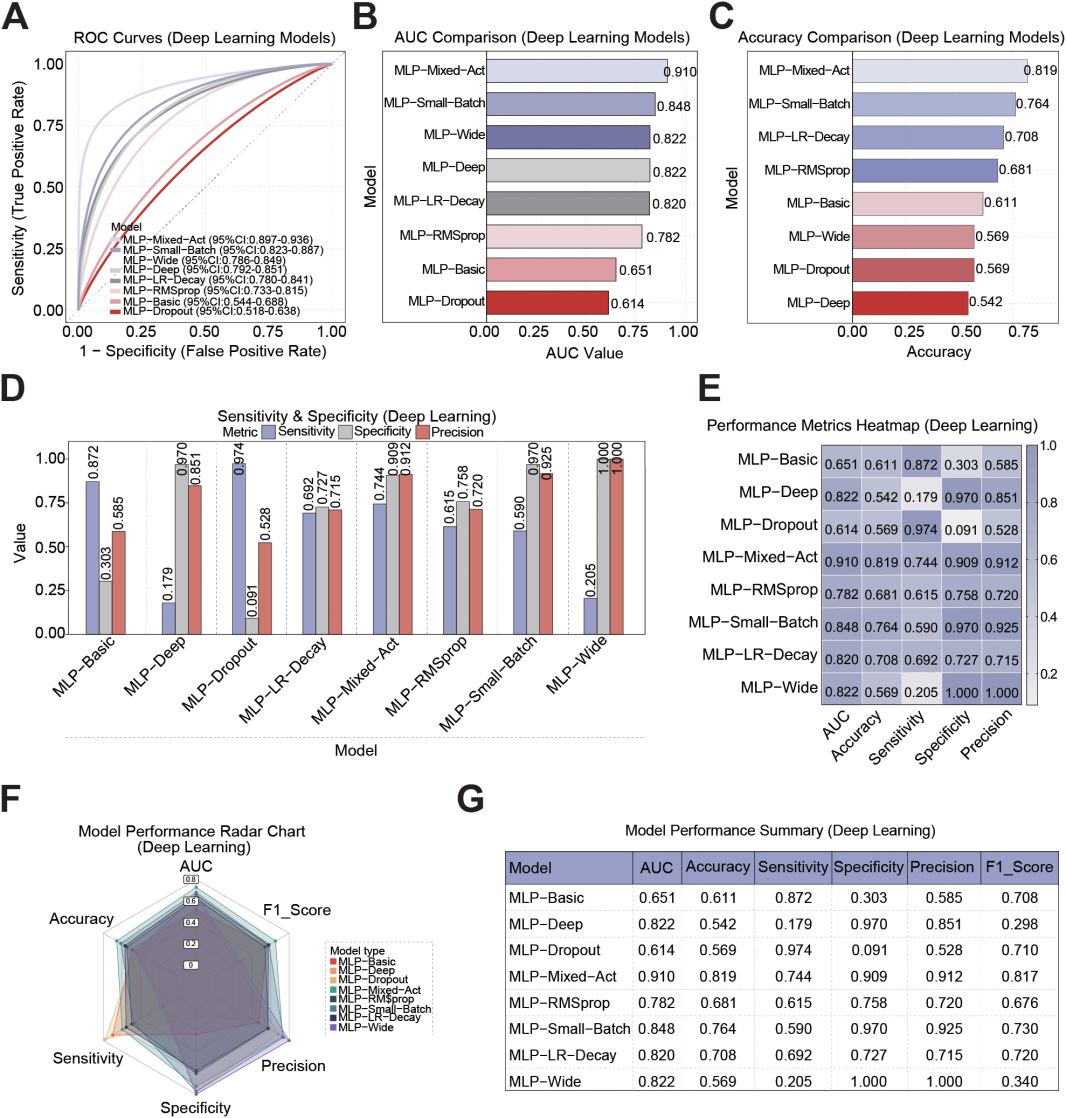
Performance comparison of DL Models in PCa recurrence prediction. **(A)** ROC curves of the DL models. **(B)** Comparison of AUC values for the DL models. **(C)** Comparison of the accuracy of the DL models. **(D)** Sensitivity, specificity and precision of each DL model in identifying recurrent and non-recurrent samples. **(E)** Performance summary heatmap of five key indicators: AUC, accuracy, sensitivity, specificity, precision, and F1 score of the DL models. The color gradient ranges from light (low) to dark (high) to reflect numerical changes in these indicators. **(F)** Performance radar chart of each DL model. **(G)** Summary table of DL model performance.

Next, we compared the predictive performance of eight DL models (MLP-Mixed-Act, MLP-Small-Batch, MLP-Wide, MLP-Deep, MLP-Basic, MLP-Dropout, and MLP-RMSprop). ROC curve analysis shows that the MLP-Mixed-Act model performs the best, with its curve closest to the upper left corner (**Figure 5A**). The comparison of AUC values further indicates that the MLP-Mixed-Act model has the strongest discriminative ability at 0.910, while the MLP-Small-Batch, MLP-Wide, and MLP-Deep models also achieved higher AUC values of 0.822, 0.822, and 0.820, respectively. In contrast, the AUC values of MLP-Basic and MLP-Dropout were 0.651 and 0.614, respectively, approaching the level of random classification (**Figure 5B**). In terms of accuracy, the MLP-Mixed-Act model also ranks first, with an accuracy of 0.819. The MLP-Small-Batch model is followed by the MLP-Wide model, with accuracy values of 0.764 and 0.744, which are better than other models (**Figure 5C**). The sensitivity, specificity and precision evaluation results showed that MLP-Mixed-Act achieved a good balance between sensitivity and specificity, with a sensitivity of 0.744, specificity of 0.909 and precision of 0.912. Although the MLP-Wide model exhibits perfect specificity and precision, its sensitivity is only 0.205. On the contrary, the MLP-Deep and MLP-Basic models showed high specificity values of 0.970 and 0.872, respectively, but extremely low sensitivity (both below 0.31), indicating limited ability to identify recurrent cases (**Figure 5D**).

We used heat maps to present the comprehensive performance results of the DL models. The results showed that MLP-Mixed-Act performed well in all indicators (AUC, accuracy, sensitivity, specificity, precision and F1 score). MLP-Small-Batch and MLP-RMSprop also demonstrated stable performance in multiple indicators. MLP-Dropout and MLP-Basic performed the worst in sensitivity and F1 score analysis (**Figure 5E**). **Figure 5F **radar map presents this result more intuitively. The MLP-Mixed-Act model shows the largest coverage area in five dimensions, followed by the MLP-Small-Batch and MLP-RMSprop models. **Figure 5G** summarizes the detailed metric values of each model. These results all indicated that the MLP-Mixed-Act model scored the highest in terms of AUC (0.910), accuracy (0.819), sensitivity (0.744), specificity (0.909), precision (0.912) and F1 score (0.817), demonstrating the best comprehensive predictive ability and being the optimal model in this study.

### 3.5 Feature importance in DL models

**Figure 6:**
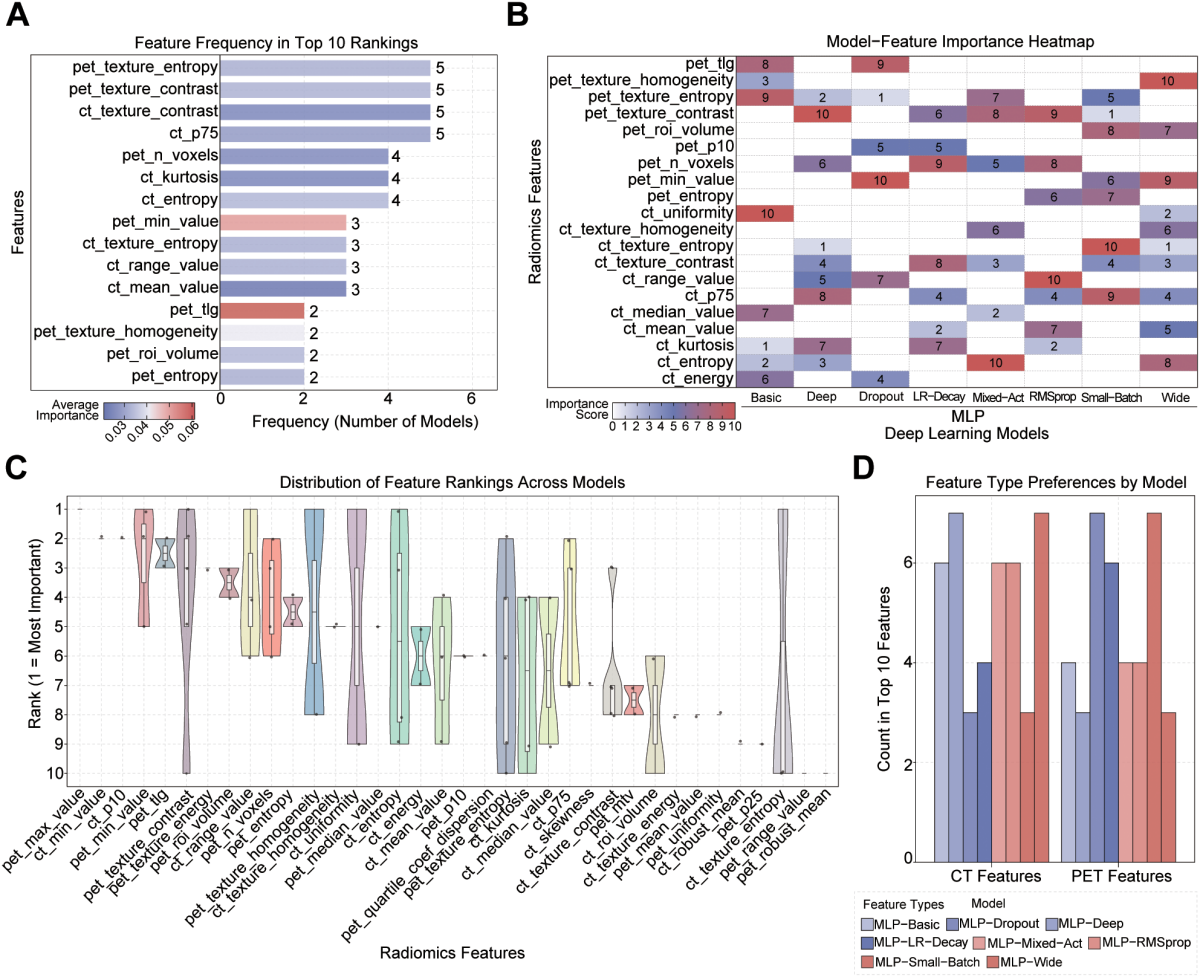
Importance analysis and preference comparison of features in DL models. **(A)** Feature frequency statistics. The color of each bar represents the average ranking of the feature in the selected model, with darker colors (red) indicating higher frequency. **(B)** Feature importance heatmap, where darker colors (red) correspond to greater importance. **(C)** Distribution of feature rankings across different models. Features are ranked by median, with the most stable and important features positioned at the top. **(D)** Preference of different DL models for feature types (CT vs PET).

Similarly, we analyzed the feature contribution in DL model recurrence prediction, and the results are shown in **Figure 6**. Overall, pet_texture_stropy, pet_textule_comtrast, ct_texture_comtrast, and ct_p75 appear frequently in multiple DL models, demonstrating their importance (**Figure 6A**). The importance heatmap of high-frequency features in different DL models (**Figure 6B**) further reveals the differences in feature dependency between DL models. The results showed that pet_texture_comtrast and pet_cin_ralue ranked high in the MLP-Deep, MLP-Dropout, and MLP_Wild models, while ct_uniformity and ct_detropy had greater weights in the MLP-Basic, MLP-Mixed-Act, and MLP-Small-Batch models. Further analysis of the stability of feature ranking (**Figure 6C**) shows that pet_texture_stropy, pet_tlg, and pet_invalue exhibit highly consistent rankings among models, indicating that they play an important role in most DL models. In contrast, ct_texture-entropy and pet_invalue are only sensitive to specific models. Finally, the preferences of CT/PET feature types for different DL models were analyzed (**Figure 6D**), and the results showed that the overall dependence of DL models on PET and CT features was balanced.

Based on the above results, in the prediction of PCa recurrence, the MLP-Mixed-Act model has the best performance, surpassing all other ML and DL models including GLMNet, demonstrating reliability and application potential. The feature preference analysis of different models also plays a crucial role in the subsequent model optimization.

## 4 Discussion

Prostate cancer is among the most common malignant neoplasms arising in the male genitourinary system, and the incidence is still rising in China. For patients with localized prostate cancer treated with radical prostatectomy, the recurrence rate is unacceptably high, with a median survival time of only two years, indicating an extremely poor prognosis with a significant treatment burden (19, 20).Timely, precise prediction of recurrence is essential in order to create personalized clinical management strategies along the continuum of care focused on timed clinical options emphasizing maximizing quality of life. Previous studies have identified a number of prognostic factors that are well recognized risk factors for recurrence after radical treatment, including tumor invasion of the peritoneum, seminal vesicle involvement, and Gleason score (21, 22). Despite the factors discussed, and the prognostic validity of each factor, predicting recurrence remains difficult due to understanding the underlying mechanisms. Therefore, more precise and effective means of early assessment of biochemical recurrence is needed to effectively guide personalized patient clinical management.

Conventional imaging modalities have limited sensitivity for early recurrence monitoring. ^18^F-PSMA-1007 PET/CT has, in turn, substantially increased the ability to detect recurrent lesions, due to its excellent localization abilities even at low PSA levels (23, 24). However, in spite of emerging research, leveraging fully quantitative characteristics in PSMA PET/CT imaging and developing individualized recurrence risk prediction tools using artificial intelligence has proven challenging. In recent years, more and more studies based on PSMA PET/CT, have begun to evaluate the risk of postoperative recurrence. Some studies have found that certain indicators of PSMA PET/CT, such as SUVmax, lesion volume, or total PSMA uptake of the lesion, are significantly correlated with biochemical recurrence and may have advantages over traditional clinical and pathological factors in early detection of recurrence (25). There are also studies attempting to combine PET/CT results with clinical variables to establish risk stratification models. The results showed that the predictive performance of this joint model is usually better than relying solely on clinical indicators, but such methods often only use a small number of manually selected image features.

Radiomics is an emerging approach in medical imaging analysis that evaluates the microscopic heterogeneity of tumors that is often undetectable to the human eye by computing a vast number of quantitative features from images.At present, radiology models based on ML and DL have been used in many studies, mainly for pathological grading, risk stratification, and prediction of treatment response and metastasis of PCa (26–28). Some PET/CT studies based on radiomics have shown that compared with traditional pet parameters, high-dimensional texture features reflecting tumor heterogeneity have certain advantages in predicting the prognosis of PCA, so relevant studies are gradually increasing (29–31). However, most of these studies only use a single modeling method, or only focus on a certain kind of algorithm in ML or DL, and the comparison between different algorithms and feature selection is still less.

Given this background, we systematically assessed and compared the performance of 10 mainstream ML models and 8 different structured DL models for predicting postoperative recurrence of PCa while also assessing the importance of PET/CT radiomics features in model development. In contrast, our radiomics artificial intelligence research systematically evaluated multiple machine learning and deep learning models, and introduced more comprehensive PET/CT derived features to more comprehensively analyze model performance, feature importance, and applicability in personalized postoperative recurrence prediction. By methodically comparing model performance across multiple metrics, namely AUC, accuracy, sensitivity, specificity, precision, F1 score, and AUC with 10-fold cross-validation, we aimed to identify a model with the best balance for generalizability and accuracy outcomes. We also aimed to identify important imaging features with consistent predictive value across multiple algorithms in order to provide a theoretical basis for the development of scalable clinical prediction tools.

Among all ML models, LDA has the highest AUC, reaching 0.951, indicates its strong ability to distinguish between recurrent and non recurrent prostate cancer cases. However, the AUC of the model in 10 fold cross validation was only 0.446, indicating insufficient generalization ability. This result indicates that the model performs well on the current dataset, but its predictability on unknown data is unknown. Similarly, although Naive Bayes, KNN, and SVM are widely used in cancer recurrence prediction, they performed poorly in this study with low AUC and accuracy. In contrast, GLMNet performed more stably, finding a good balance between AUC and CV AUC. GLMNet model performs particularly evenly, with a model AUC of 0.818, an accuracy of 0.736, a sensitivity of 0.769, a specificity of 0.697, a precision of 0.731 and a 10 fold CV AUC of 0.702, indicating its good generalization ability. Compared to models based on tree structure or distance measurement, GLMNet model can effectively reduce the risk of overfitting when processing high-dimensional radiomics features by introducing regularization mechanisms. Therefore, it has advantages in research scenarios with relatively limited sample sizes and is more in line with practical clinical application needs. Feature importance analysis shows that in most machine learning models, PET texture features are ranked higher, indicating that these features have good stability and universality in different models. Specifically, parameters such as pet_entropy and pet_uniformity can reflect the heterogeneity and metabolic complexity within tumors, which are often closely related to the invasiveness and recurrence risk of tumors. This also explains why they can maintain relatively robust predictive value in different algorithms. Although CT features such as ct_uniformity and ct_deergy are also considered, their contribution to the model is not stable, further highlighting the potential advantages of PET imaging in predicting recurrence based on ML models.

Among the DL models, the MLP-Mixed-Act model yielded the best performance overall with AUC of 0.910, accuracy of 0.819, sensitivity of 0.744, specificity of 0.909, precision of 0.912 and F1 score of 0.817. The use of mixed activation functions may enhance the model’s representation of nonlinear features, improve learning stability, and more effectively integrate heterogeneous features of PET and CT radiology. In a similar fashion, the mixed activation function architecture showed relatively similar classification performance and had improved generalization properties. In contrast, the results of the MLP-Basic and MLP-Dropout models exhibited both low sensitivity and low F1 score suggesting that the features were not effective in identifying all cases of recurrence. This may be related to the insufficiently complex network structure of the model, or excessive regularization, which limits the model’s learn ability effectively from radiomics features in small sample environments. Lastly, the importance analysis of features showed that the MLP-Mixed-Act was able to balance the features of PET and CT together to the maximum benefit of the models, as the combinations of features alone appeared that either feature limited the overall prediction capability. In terms of feature importance, PET features are once again confirmed as key factors in most DL models. Features such as pet_texture_entropy and pet_texture_strass often appear in the model, consistent with the results of ML models. However, unlike ML models, deep learning models have a more balanced dependence on PET and CT features, although models with different architectures show different preferences for various types of features.

Comparing ML and DL models together, it can be seen that the choice of modeling method largely depends on the characteristics of the data itself and the research objectives. In ML models, methods such as LDA and DT have simple structures and relatively easy to interpret results, but their generalization ability is limited. In contrast, GLMNet, as a more complex model, is more robust in cases of large data changes and exhibits stronger generalization ability. On the other hand, DL models have an advantage in capturing complex nonlinear relationships. MLP-Mixed-Act model is significantly ahead in accuracy and discriminative ability, indicating that it can provide more detailed and powerful predictions in high-dimensional and large-scale data. However, such models typically require more data and computing resources, and have poorer interpretability compared to ML models.

In conclusion, this was the first study to systematically evaluate the performance of a total of 10 ML and 8 DL models for predicting postoperative recurrence of PCa. Our study offered a careful evaluation of prediction performance, generalizability, and feature dependency for each model, and highlighted the distinctive benefits of several of models, including MLP-Mixed-Act. We then established stable, key features, such as pet_detropy, pet_p10, and pet_uniformity, from our feature importance analysis. These features will be the basis for future refinement of the prediction model. The main discovery of the study shows that radiomics features derived from ^18^F-PSMA PET/CT and AI models can significantly enhance the accuracy of recurrence risk prediction, and identified the MLP-Mixed-Act model, providing a potential approach for developing precise and personalized treatment for PCa recurrence. As non-invasive, scalable tools, the approach has significant potential to aid in postoperative patient risk-stratification and post-operative follow-seriation, while being a significant step toward individualized, structured, and intelligent development of the diagnosis and treatment of PCa.

## 5 Limitations

While the findings of this research offer some supportive but early evidence of radiomics-based machine learning and deep learning prediction tools, there are limitations. First, since this study was retrospective in design, patient selection bias was potentially introduced which could limit the study’s comprehensiveness. Second, the relatively small sample size could compromise the stability and generalizability of the model. Third, without independent external validation, this limits the ability to validate the model beyond the original data set.

In the future, we will collect more samples from more centers to further optimize the model and enhance its predictive ability. At the same time, we will also incorporate other clinical indicators such as PSA levels and Gleason scores to improve the accuracy of the model. Our goal is to provide more precise and practical guidance for developing personalized treatment strategies. In addition to predictive performance, the clinical applicability of recurrence risk prediction models is also crucial. Early identification of patients with high biochemical recurrence risk can directly affect treatment decisions. Combining radiomics based predictions with traditional risk factors such as Gleason score can help more accurately stratify postoperative risks.

## Data Availability

The original contributions presented in the study are included in the article/supplementary material. Further inquiries can be directed to the corresponding authors.

## References

[1] Sung H Ferlay J Siegel RL Laversanne M Soerjomataram I Jemal A . Global cancer statistics 2020: Globocan estimates of incidence and mortality worldwide for 36 cancers in 185 countries. CA Cancer J Clin. (2021) 71:209–49. doi: 10.3322/caac.21660. PMID: 33538338

[2] Lin X Kapoor A Gu Y Chow MJ Xu H Major P . Assessment of biochemical recurrence of prostate cancer (review). Int J Oncol. (2019) 55:1194–212. doi: 10.3892/ijo.2019.4893. PMID: 31638194 PMC6831208

[3] Van den Broeck T van den Bergh RCN Arfi N Gross T Moris L Briers E . Prognostic value of biochemical recurrence following treatment with curative intent for prostate cancer: A systematic review. Eur Urol. (2019) 75:967–87. doi: 10.1016/j.eururo.2018.10.011. PMID: 30342843

[4] Zeng T Xie Y Chai K Sang H . The application of prostate specific membrane antigen in the diagnosis and treatment of prostate cancer: Status and challenge. Onco Targets Ther. (2024) 17:991–1015. doi: 10.2147/OTT.S485869. PMID: 39564453 PMC11573878

[5] Wang F Li Z Feng X Yang D Lin M . Advances in psma-targeted therapy for prostate cancer. Prostate Cancer Prostatic Dis. (2022) 25:11–26. doi: 10.1038/s41391-021-00394-5. PMID: 34050265

[6] Bouchelouche K Choyke PL . Advances in prostate-specific membrane antigen pet of prostate cancer. Curr Opin Oncol. (2018) 30:189–96. doi: 10.1097/CCO.0000000000000439. PMID: 29465429 PMC6003670

[7] Papp L Spielvogel CP Grubmuller B Grahovac M Krajnc D Ecsedi B . Supervised machine learning enables non-invasive lesion characterization in primary prostate cancer with [ (68)Ga]Ga-psma-11 pet/mri. Eur J Nucl Med Mol Imaging. (2021) 48:1795–805. doi: 10.1007/s00259-020-05140-y. PMID: 33341915 PMC8113201

[8] Jiang J Chen L Ji X Zheng X Hong J Tang K . ((18)F)-psma-1007pet/ct in patients with biochemical recurrence after radical prostatectomy: Diagnostic performance and impact on treatment management. Res Diagn Interv Imaging. (2023) 5:100021. doi: 10.1016/j.redii.2022.100021. PMID: 39076163 PMC11265200

[9] Xie Y Zhao H Guo Y Meng F Liu X Zhang Y . A pet/ct nomogram incorporating suvmax and ct radiomics for preoperative nodal staging in non-small cell lung cancer. Eur Radiol. (2021) 31:6030–8. doi: 10.1007/s00330-020-07624-9. PMID: 33560457 PMC8270849

[10] Feretzakis G Juliebo-Jones P Tsaturyan A Sener TE Verykios VS Karapiperis D . Emerging trends in ai and radiomics for bladder, kidney, and prostate cancer: A critical review. Cancers (Basel). (2024) 16. doi: 10.3390/cancers16040810. PMID: 38398201 PMC10886599

[11] Pak S Park SG Park J Cho ST Lee YG Ahn H . Applications of artificial intelligence in urologic oncology. Investig Clin Urol. (2024) 65:202–16. doi: 10.4111/icu.20230435. PMID: 38714511 PMC11076794

[12] Piran Nanekaran N Felefly TH Schieda N Morgan SC Mittal R Ukwatta E . Prediction of prostate cancer recurrence after radiotherapy using a fused machine learning approach: Utilizing radiomics from pretreatment t2w mri images with clinical and pathological information. BioMed Phys Eng Express. (2024). doi: 10.1088/2057-1976/ad8201. PMID: 39353461

[13] Wang H Wang K Zhang Y Chen Y Zhang X Wang X . Deep learning-based radiomics model from pretreatment adc to predict biochemical recurrence in advanced prostate cancer. Front Oncol. (2024) 14:1342104. doi: 10.3389/fonc.2024.1342104. PMID: 38476369 PMC10928490

[14] Tapper W Carneiro G Mikropoulos C Thomas SA Evans PM Boussios S . The application of radiomics and ai to molecular imaging for prostate cancer. J Pers Med. (2024) 14:287. doi: 10.3390/jpm14030287. PMID: 38541029 PMC10971024

[15] Li W Hu R Zhang Q Yu Z Deng L Zhu X . Artificial intelligence in prostate cancer. Chin Med J (Engl). (2025) 138:1769–82. doi: 10.1097/CM9.0000000000003689. PMID: 40629505 PMC12321470

[16] Ejaz ZH Shaikh RH Fatimi AS Khan SR . Unlocking artificial intelligence, machine learning and deep learning to combat therapeutic resistance in metastatic castration-resistant prostate cancer: a comprehensive review. Ecancermedicalscience. (2025) 19:1953. doi: 10.3332/ecancer.2025.1953. PMID: 40949469 PMC12426504

[17] Zhong J Davey A Frood R McWilliam A Shortall J Reardon M . Combining mri radiomics, hypoxia gene signature score and clinical variables for prediction of biochemical recurrence-free survival after radiotherapy in prostate cancer. Radiol Med. (2025) 130:1139–48. doi: 10.1007/s11547-025-02037-4. PMID: 40601075 PMC12367909

[18] Paquier Z Chao SL Acquisto A Fenton C Guiot T Dhont J . Radiomics software comparison using digital phantom and patient data: IBSI-compliance does not guarantee concordance of feature values. BioMed Phys Eng Express. (2022) 8. doi: 10.1088/2057-1976/ac8e6f. PMID: 36049399

[19] Jiang Q Xie M He M Yan F Chen M Xu S . Pitx2 methylation: A novel and effective biomarker for monitoring biochemical recurrence risk of prostate cancer. Med (Baltimore). (2019) 98:e13820. doi: 10.1097/MD.0000000000013820. PMID: 30608394 PMC6344153

[20] Kruczek K Ratterman M Tolzien K Sulo S Lestingi TM Nabhan C . A phase ii study evaluating the toxicity and efficacy of single-agent temsirolimus in chemotherapy-naive castration-resistant prostate cancer. Br J Cancer. (2013) 109:1711–6. doi: 10.1038/bjc.2013.530. PMID: 24008662 PMC3790181

[21] Rezaee ME Pallauf M Fletcher SA Han M Pavlovich CP Cornelia Ding CK . Risk of biochemical recurrence in patients with grade group 1 prostate cancer with extraprostatic extension treated with radical prostatectomy. J Urol. (2024) 211:407–14. doi: 10.1097/JU.0000000000003825. PMID: 38109699

[22] Cimitan M Evangelista L Hodolic M Mariani G Baseric T Bodanza V . Gleason score at diagnosis predicts the rate of detection of 18f-choline pet/ct performed when biochemical evidence indicates recurrence of prostate cancer: Experience with 1, 000 patients. J Nucl Med. (2015) 56:209–15. doi: 10.2967/jnumed.114.141887. PMID: 25552670

[23] Armany D Vo L Self D Baskaranathan S Hossack T Bariol S . The role of 18f psma-1007 pet/ct in the staging and detection of recurrence of prostate cancer, a scoping review. Cancers (Basel). (2025) 17. doi: 10.3390/cancers17061049. PMID: 40149383 PMC11941518

[24] Abrahamsen BS Tandstad T Aksnessaether BY Bogsrud TV Castillejo M Hernes E . Added value of [18f]psma-1007 pet/ct and pet/mri in patients with biochemically recurrent prostate cancer: Impact on detection rates and clinical management. J Magn Reson Imaging. (2025) 61:466–77. doi: 10.1002/jmri.29386. PMID: 38679841 PMC11645485

[25] Roberts MJ Maurer T Perera M Eiber M Hope TA Ost P . Using PSMA imaging for prognostication in localized and advanced prostate cancer. Nat Rev Urol. (2023) 20:23–47. doi: 10.1038/s41585-022-00670-6. PMID: 36473945

[26] Michaely HJ Aringhieri G Cioni D Neri E . Current value of biparametric prostate mri with machine-learning or deep-learning in the detection, grading, and characterization of prostate cancer: A systematic review. Diagnostics (Basel). (2022) 12. doi: 10.3390/diagnostics12040799. PMID: 35453847 PMC9027206

[27] Rodrigues NM Almeida JG Rodrigues A Vanneschi L Matos C Lisitskaya MV . Deep learning features can improve radiomics-based prostate cancer aggressiveness prediction. JCO Clin Cancer Inform. (2024) 8:e2300180. doi: 10.1200/CCI.23.00180. PMID: 39292984

[28] Ogulmus FE Almalioglu Y Tamam MO Yildirim B Uysal E Numanoglu C . Integrating pet/ct, radiomics and clinical data: An advanced multi-modal approach for lymph node metastasis prediction in prostate cancer. Comput Biol Med. (2025) 184:109339. doi: 10.1016/j.compbiomed.2024.109339. PMID: 39522134

[29] Ha S Choi H Paeng JC Cheon GJ . Radiomics in oncological PET/CT: a methodological overview. Nucl Med Mol Imaging. (2019) 53:14–29. doi: 10.1007/s13139-019-00571-4. PMID: 30828395 PMC6377580

[30] Unger M Kather JN . Deep learning in cancer genomics and histopathology. Genome Med. (2024) 16:44. doi: 10.1186/s13073-024-01315-6. PMID: 38539231 PMC10976780

[31] Kleiburg F de Geus-Oei LF Spijkerman R Noortman WA van Velden FHP Manohar S . Baseline PSMA PET/CT parameters predict overall survival and treatment response in metastatic castration-resistant prostate cancer patients. Eur Radiol. (2025) 35:4223–32. doi: 10.1007/s00330-025-11360-3. PMID: 39843627 PMC12165979

