# Supplementary material for: Predicting recurrence of prostate cancer after radical treatment using AI models based on PET/CT radiomics: a dual-center study
Source: Front Oncol. 2026 Apr 6;16:1733046. doi: 10.3389/fonc.2026.1733046 (PMC13093974; doi:10.3389/fonc.2026.1733046)

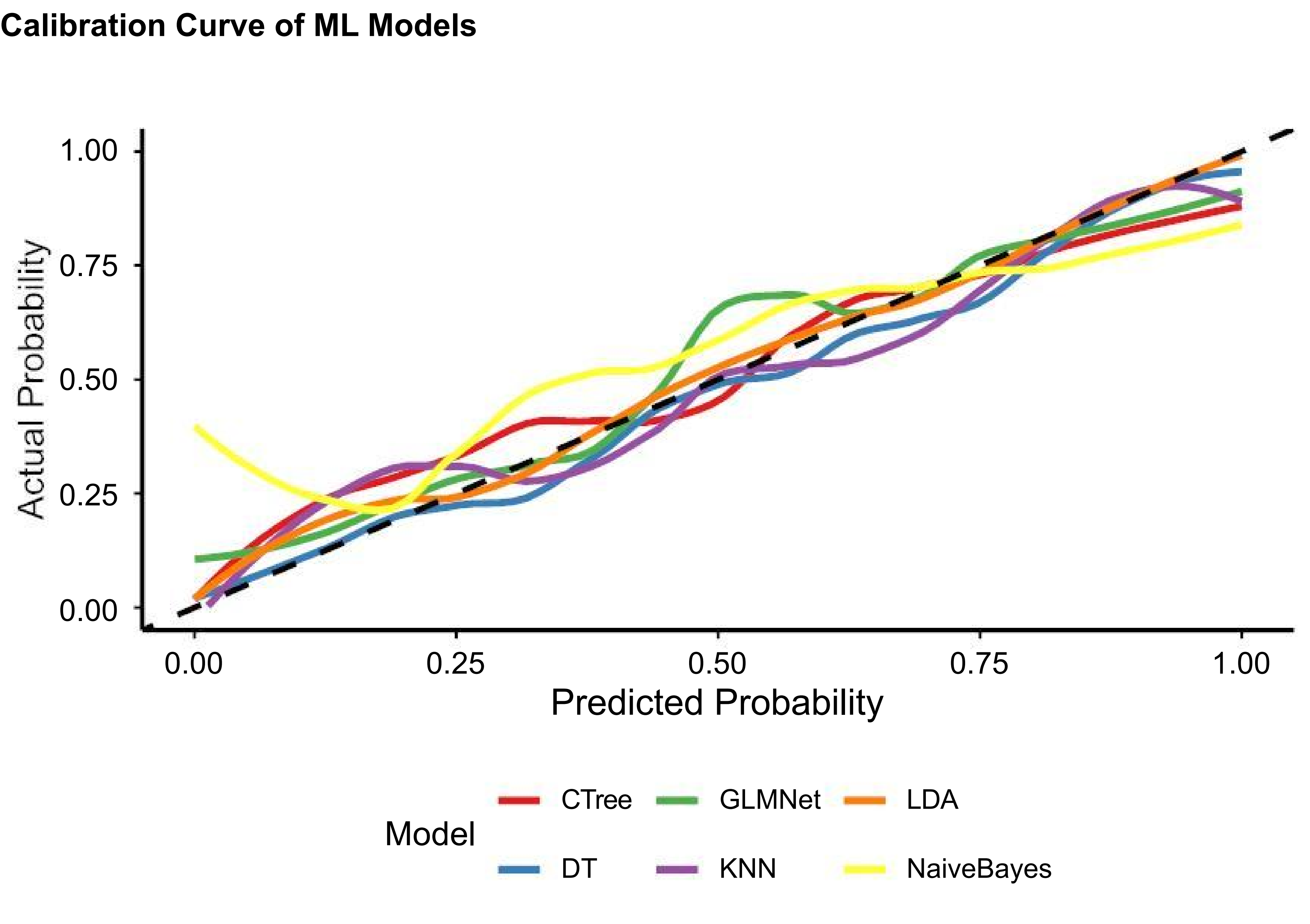

Calibration Curve of ML Models
1.00
0.75
0.50
0.25
0.00
Actual Probability
0.00
0.25
0.50
0.75
1.00
Predicted Probability
Model
CTree
GLMNet
LDA
DT
KNN
NaiveBayes

Decision Curve Analysis of ML Models

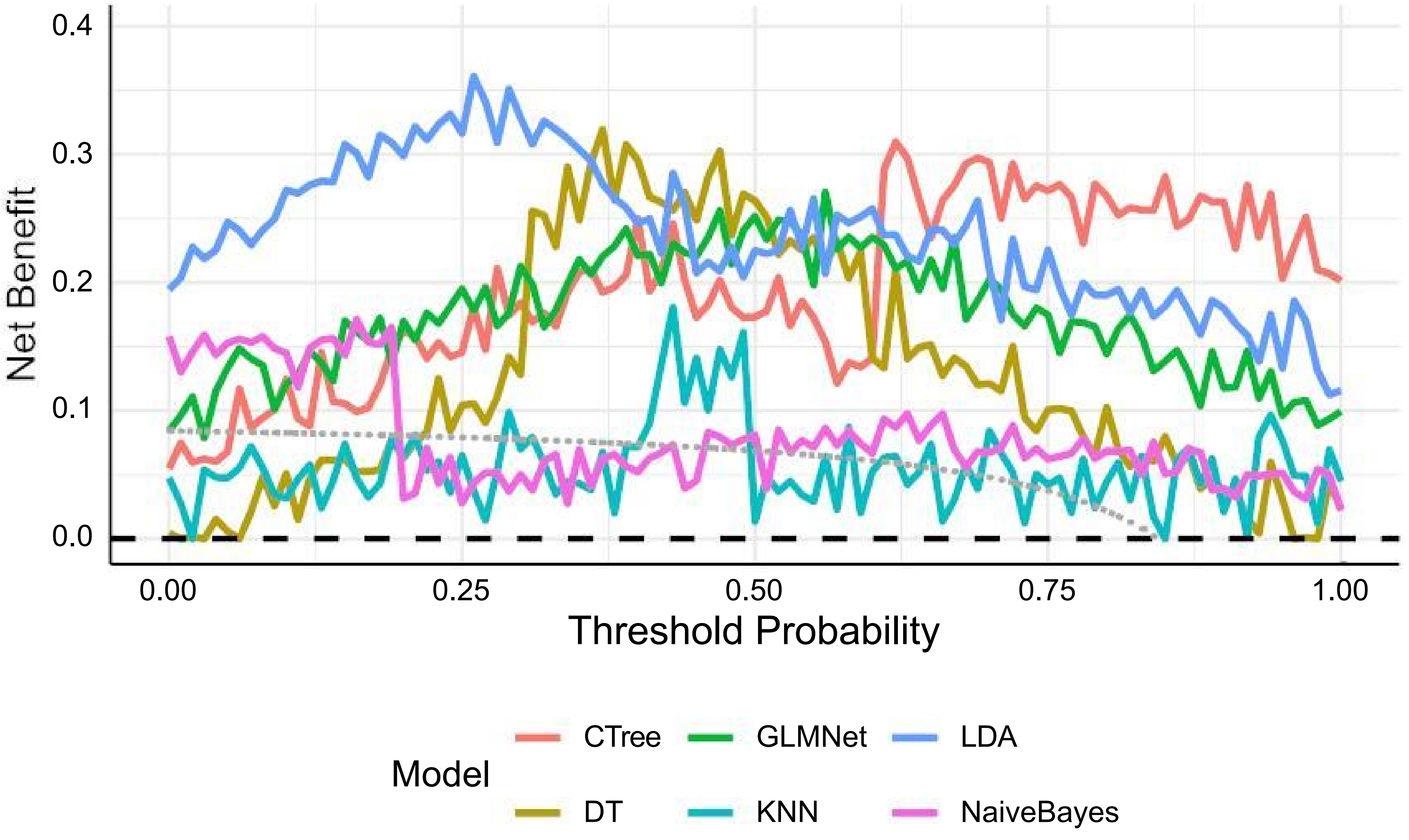

Calibration Curve of DL Models

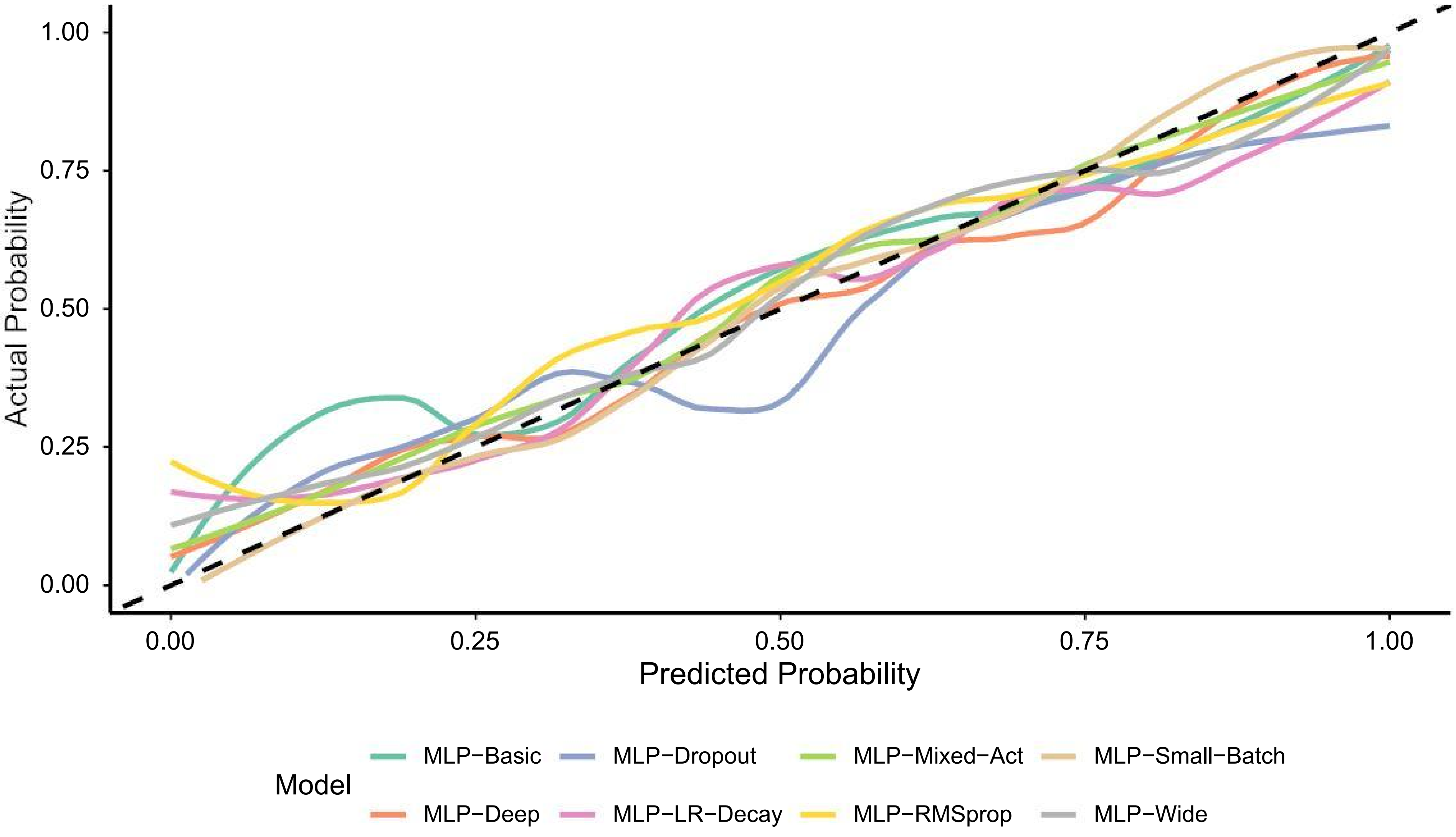

Decision Curve Analysis of DL Models

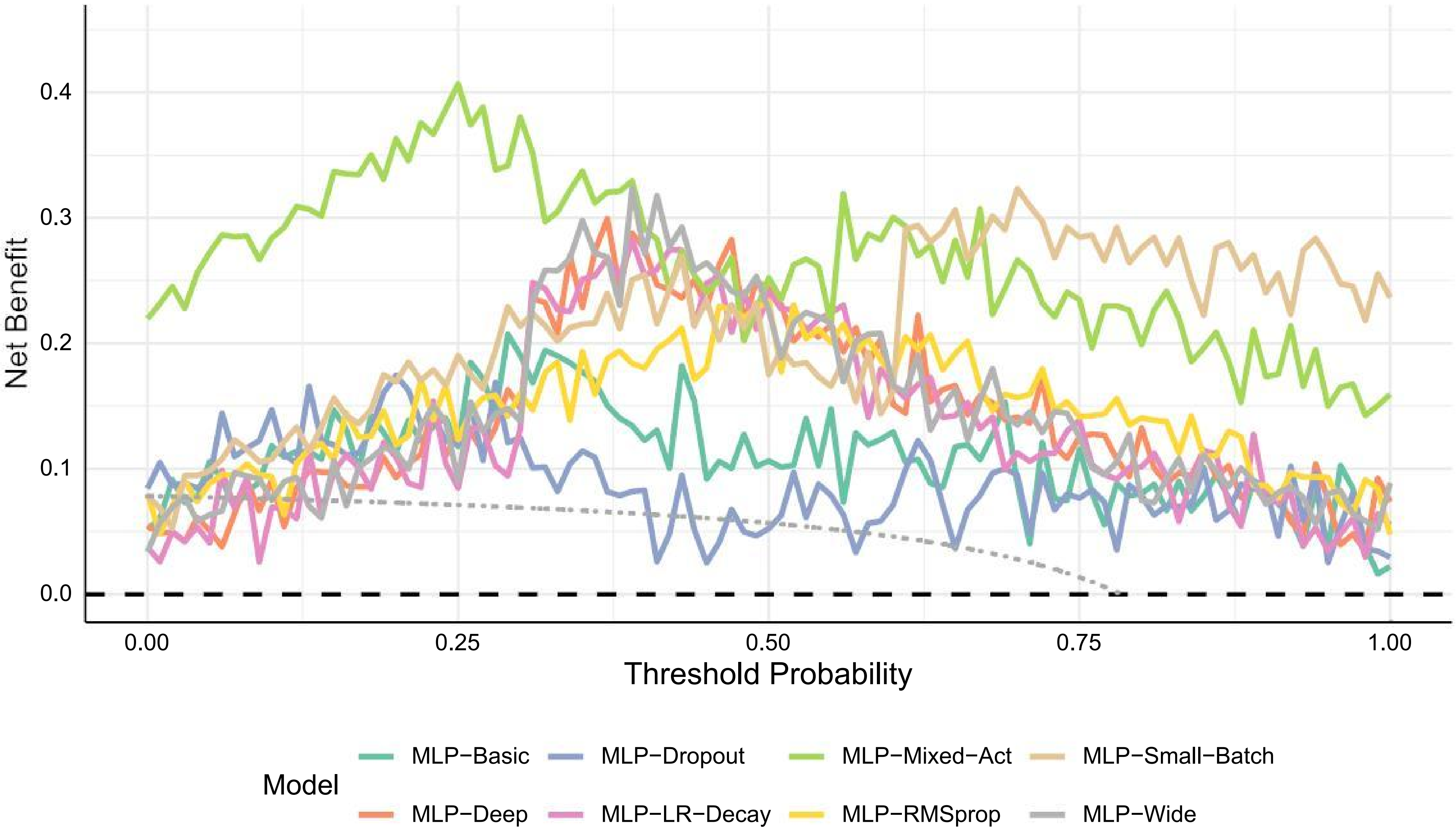

Supplement: Supplementary file 1 [file DataSheet1.pdf]
