# Supplementary material for: Predicting recurrence of prostate cancer after radical treatment using AI models based on PET/CT radiomics: a dual-center study
Source: Front Oncol. 2026 Apr 6;16:1733046. doi: 10.3389/fonc.2026.1733046 (PMC13093974; doi:10.3389/fonc.2026.1733046)
Supplement: Supplementary file 2 [file Table1.docx]

**Table 1. PET/CT的特征提取信息对比**

| Characteristics | Non-recurrence(N=33) | Recurrence(N=39) | P.value |
| --- | --- | --- | --- |
| ct_n_voxels | 16350.97±8622.14 | 16458.62±19084.53 | 0.49 |
| ct_mean_value | 57.00±29.29 | 52.25±18.34 | 0.21 |
| ct_sd_value | 68.91±61.84 | 48.62±49.78 | 0.07 |
| ct_median_value | 43.82±9.91 | 43.67±5.00 | 0.47 |
| ct_min_value | -63.06±45.95 | -51.95±39.55 | 0.14 |
| ct_max_value | 645.76±531.79 | 475.15±481.76 | 0.08 |
| ct_range_value | 708.82±531.98 | 527.10±501.53 | 0.07 |
| ct_p10 | 18.09±27.32 | 24.46±5.18 | 0.10 |
| ct_p25 | 30.39±22.53 | 34.62±4.97 | 0.15 |
| ct_p75 | 55.70±9.44 | 52.87±6.51 | 0.08 |
| ct_p90 | 99.39±89.21 | 77.20±65.91 | 0.12 |
| ct_roi_volume | 16350.97±8622.14 | 16458.62±19084.53 | 0.49 |
| ct_skewness | 2.98±3.68 | 1.94±3.12 | 0.10 |
| ct_kurtosis | 31.86±58.69 | 17.94±27.50 | 0.11 |
| ct_coefficient_variation | 1.69±5.47 | 0.78±0.58 | 0.17 |
| ct_quartile_coef_dispersion | 0.42±1.98 | 0.21±0.05 | 0.27 |
| ct_energy | 196050972.76±265510631.96 | 180433670.51±388712076.06 | 0.42 |
| ct_entropy | 6.36±0.64 | 6.06±0.51 | 0.02* |
| ct_robust_mean | 44.64±14.83 | 44.40±6.89 | 0.47 |
| ct_mad | 17.43±9.93 | 13.38±2.79 | 0.01* |
| ct_uniformity | 0.02±5.2e-3 | 0.02±4.6e-3 | 0.01* |
| ct_texture_contrast | 3.35±1.46 | 3.28±0.91 | 0.41 |
| ct_texture_energy | 0.29±0.20 | 0.23±0.17 | 0.10 |
| ct_texture_homogeneity | 0.25±0.08 | 0.25±0.06 | 0.32 |
| ct_texture_entropy | 1.87±0.76 | 2.05±0.72 | 0.15 |
| pet_n_voxels | 4538.33±3113.36 | 3169.49±2884.36 | 0.03* |
| pet_mean_value | 4745.19±2614.75 | 8320.57±12170.99 | 0.04* |
| pet_sd_value | 1593.80±947.93 | 4457.22±9162.56 | 0.03* |
| pet_median_value | 4723.23±2539.75 | 7602.07±11966.72 | 0.08 |
| pet_min_value | 309.92±494.54 | 837.21±1174.69 | 0.01* |
| pet_max_value | 11298.85±6422.58 | 24183.93±39468.68 | 0.03* |
| pet_range_value | 10988.93±6103.48 | 23346.73±39023.47 | 0.03* |
| pet_p10 | 2738.04±1845.07 | 3491.25±2886.20 | 0.09 |
| pet_p25 | 3816.46±2198.13 | 4904.51±4381.38 | 0.09 |
| pet_p75 | 5708.98±3100.22 | 11106.89±18750.79 | 0.04 |
| pet_p90 | 6721.71±3743.26 | 14040.94±24269.26 | 0.04 |
| pet_roi_volume | 4538.33±3113.36 | 3169.49±2884.36 | 0.03* |
| pet_skewness | 0.04±0.48 | 0.76±2.21 | 0.03* |
| pet_kurtosis | 0.70±0.77 | 7.50±36.22 | 0.12 |
| pet_coefficient_variation | 0.34±0.08 | 0.39±0.20 | 0.08 |
| pet_quartile_coef_dispersion | 0.21±0.08 | 0.23±0.17 | 0.18 |
| pet_energy | 161818440137.66±223305611107.88 | 256372978239.67±836819031593.00 | 0.25 |
| pet_entropy | 10.05±0.78 | 9.20±1.31 | 0.00* |
| pet_robust_mean | 4739.91±2589.86 | 7991.42±11565.74 | 0.05* |
| pet_mad | 1332.71±873.47 | 3971.98±11249.51 | 0.08 |
| pet_uniformity | 1.3e-3±1.1e-3 | 3.1e-3±4.0e-3 | 0.01* |
| pet_suv_mean | 4745.19±2614.75 | 8320.57±12170.99 | 0.04* |
| pet_suv_max | 11298.85±6422.58 | 24183.93±39468.68 | 0.03* |
| pet_suv_peak | 7366.02±4153.74 | 16032.69±27460.29 | 0.03* |
| pet_tlg | 23426707.20±21741223.93 | 16914319.15±22705336.77 | 0.11 |
| pet_mtv | 4538.33±3113.36 | 3169.49±2884.36 | 0.03* |
| pet_texture_contrast | 4.72±0.97 | 4.83±1.62 | 0.36 |
| pet_texture_energy | 0.07±0.01 | 0.10±0.07 | 0.03* |
| pet_texture_homogeneity | 0.18±0.03 | 0.18±0.05 | 0.32 |
| pet_texture_entropy | 2.85±0.15 | 2.72±0.38 | 0.03* |
